# Engineering Viroid Resistance

**DOI:** 10.3390/v7020634

**Published:** 2015-02-10

**Authors:** Athanasios Dalakouras, Elena Dadami, Michael Wassenegger

**Affiliations:** 1 RLP AgroScience GmbH, AIPlanta-Institute for Plant Research, Neustadt, 67435, Germany; E-Mail: athanasios.dalakouras@agroscience.rlp.de; 2 RLP AgroScience GmbH, AIPlanta-Institute for Plant Research, Neustadt, 67435, Germany; E-Mail: elena.dadami@agroscience.rlp.de; 3 RLP AgroScience GmbH, AIPlanta-Institute for Plant Research, Neustadt, Germany and Centre for Organisational Studies (COS) Heidelberg, University of Heidelberg, Heidelberg, 69120, Germany

**Keywords:** RNA interference, artificial micro RNAs, transgenic plants

## Abstract

Viroids are non-encapsidated, non-coding, circular, single-stranded RNAs (ssRNAs). They are classified into the families *Pospiviroidae* and *Avsunviroidae*, whose members replicate in the nucleus and chloroplast of plant cells, respectively. Viroids have a wide host range, including crop and ornamental plants, and can cause devastating diseases with significant economic losses. Thus, several viroids are world-wide, classified as quarantine pathogens and, hence, there is an urgent need for the development of robust antiviroid strategies. RNA silencing-based technologies seem to be a promising tool in this direction. Here, we review the recent advances concerning the complex interaction of viroids with the host’s RNA silencing machinery, evaluate past and present antiviroid approaches, and finally suggest alternative strategies that could potentially be employed in the future in order to achieve transgenic and non-transgenic viroid-free plants.

## 1. Viroids

Viroids are non-encapsidated, non-coding single-stranded (ss), 250–400 nucleotide (nt)-long circular RNA molecules, which may cause devastating diseases in several plant species [1]. They are classified into the families *Pospiviroidae* and *Avsunviroidae*, replicating in the nucleus and chloroplast of plant cells, respectively [2,3]. *Pospiviroidae* are comprised of more than 24 species, including *Potato spindle tuber viroid* (PSTVd) (type species), Citrus *exocortis viroid* (CEVd), *Hop latent viroid* (HLVd), and *Hop stunt viroid* (HSVd). *Avsunviroidae* members are mainly restricted to *Avocado sunblotch viroid* (ASBVd), *Eggplant latent viroid* (ELVd), *Chrysanthemum chlorotic mottle viroid* (CChMVd), and *Peach latent mosaic viroid* (PLMVd) [4].

Upon entering the plant cell, *Pospiviroidae* are assumed to be transported to the nucleus by the nuclear localization signal (NLS)- and bromodomain-containing VIROID RNA-BINDING PROTEIN 1 (VIRP1) [5]. In the nucleus, they replicate by DNA-DEPENDENT RNA POLYMERASE II (POLII) [6] *via* the asymmetric rolling cycle pathway [7]. The circular monomeric ssRNA(+) is transcribed into linear multimeric ssRNA(−), which serves as the template for the production of linear multimeric ssRNA(+), which traffics into the nucleolus to be finally processed into unit-length circular RNAs by a host RNase and DNA ligase [8,9]. The mature viroid then moves to neighboring cells through plasmodesmata and to distant parts of the plant through the vascular system [10,11].

When *Avsunviroidae* enter the plant cell, it is suggested that they are transported into the nucleus before they are targeted to chloroplasts [12]. In the chloroplast, they replicate by the nucleus-encoded RNA polymerase in a symmetric replication cycle that includes a circular RNA of (−) polarity. Processing of the *Avsunviroidae* relies primarily on autocatalytic activities displayed by hammerhead ribozyme domains of RNA strands of both polarities, but host proteins like chloroplast tRNA ligase may also participate in the process [13,14].

## 2. Viroids and RNA Silencing

### 2.1. RNA Silencing

RNA silencing in plants refers to a network of pathways that are important for normal plant development, genome stability, defense against invading nucleic acids, and plastic response to biotic and abiotic stress [15]. In general, RNA silencing is induced by the presence of double stranded RNA (dsRNA). Sources of dsRNA are, among others, replication intermediates of viruses and viroids, transcription of inverted repeats, stress-induced overlapping antisense transcripts, and RNA-DIRECTED RNA POLYMERASE (RDR) transcription of sense aberrant transcripts [16]. The DICER-LIKE (DCL) endonucleases, DCL1/DCL4, DCL2, and DCL3, process dsRNA into 21-, 22- and 24-nucleotide (nt) small interfering RNAs (siRNAs), respectively [17]. Mature siRNAs exhibit 3' 2-nt overhangs and become stabilized through 3' end methylation by HUA ENHANCER1 (HEN1) [18] and, depending on their 5' terminal nucleotide, siRNAs are loaded onto specific ARGONAUTE (AGO) proteins. In general, 21-nt siRNAs are loaded onto AGO1 and mediate cleavage or translational arrest of complementary RNA in the cytoplasm, in a process termed post-transcriptional gene silencing (PTGS) [15,19]. In the nucleus, AGO4-loaded 24-nt siRNAs are involved in the targeting of *de novo* methyltransferases to cognate DNA, in a process termed RNA-directed DNA methylation (RdDM) [20,21].

### 2.2. Viroid Replication Induces Host’s RNA Silencing Machinery

Similar to viruses, viroid replication elicit the plant RNA silencing machinery [22,23]. Importantly, infection with both, the nuclear-replicating *Pospiviroidae* and the chloroplast-replicating *Avsunviroidae*, is associated with the accumulation of abundant viroid-derived siRNAs (vd-siRNAs) [24,25]. Presumably, highly structured single stranded RNA (ssRNA) molecules and dsRNA replication intermediates (and/or RDR-produced transcripts) serve as DCL substrates. Similar to endogenous siRNAs, vd-siRNAs are phosphorylated at their 5'-end, HEN1-methylated at their 3'-end [26], and loaded onto AGOs [27]. Whether AGO-loaded vd-siRNAs are present and active in the cytoplasm and in the nucleus is not clear. At least in tomato, PSTVd vd-siRNAs were found to accumulate in the cytoplasm but not in the nucleus [28]. Accordingly, PSTVd vd-siRNAs could trigger cytoplasmic but not nuclear PTGS in tobacco plants [29,30].

The molecular basis of viroid pathogenicity is not clear, but RNA silencing and the accumulation of vd-siRNAs seem to have active roles [1]. Importantly, *Pospiviroidae* replication appears to affect the DNA methylation status of host ribosomal genes [31], while both *Pospiviroidae* and *Avsunviroidae* vd-siRNAs seem to target several host mRNAs for trans-PTGS [32,33,34,35,36]. Importantly, it was recently shown that virus infection triggers widespread silencing of host genes, *via* the production of RDR1/DCL4/AGO2-dependent virus-activated siRNAs (vasiRNAs) [37]. Whether viroid infection trigger a similar mechanism is unclear, but it seems highly probable, especially when taking into consideration that RDR1 is activated upon viroid infection [38].

## 3. Initial RNA-Based Approaches to Achieve Viroid Resistance

Traditional antiviroid methods employed the screening of germplasm for possible resistance, obtaining stock plants free of viroids *via* tissue culture (meristem tip culture, stigma/style somatic embryogenesis, thermotherapy, psychrotherapy), tool disinfection, and usage of ribavirin and virazole [3]. However, as preventives, these methods offer no real resistance to viroids.

During the last two decades, RNA-based approaches have been used to obtain viroid-resistant transgenic plants. (1) In transgenic potato plants expressing antisense PSTVd RNAs of 18-nt and 173-nt, designed to hybridize to the (+) and (−) strand, respectively, viroid replication was significantly delayed [39]; (2) CEVd inoculation of transgenic tomato plants expressing ribozymes directed against the (+) and (−) strands of CEVd resulted in a moderate reduction of CEVd accumulation while, in contrast, antisense RNAs targeting either the CEVd (+) or CEVd (−) strand displayed increased CEVd RNA accumulation [40]; (3) transgenic potato plants expressing a hammerhead ribozyme targeting the PSTVd (−) strand suppressed viroid accumulation [41]; finally, (4) transgenic potato plants expressing the yeast-derived dsRNA ribonuclease pac1 (which was shown to digest PSTVd RNA *in vitro*) suppressed PSTVd infection and accumulation [42].

## 4. RNA Silencing As an Antiviroid Strategy

### 4.1. Examples Where RNA Silencing Failed to Confer Viroid Resistance

Subsequently to the discovery of RNA silencing [43] initial viroid silencing experiments employing dsRNA molecules failed to achieve the anticipated effects. Hence, while viroids were recognized to elicit the host’s RNA silencing machinery, it was challenged whether they were actually susceptible to it. Several lines of experimental evidence underlined this skepticism. (1) Vd-siRNAs of *Pospiviroidae* and *Avsunviroidae* trigger trans-PTGS of homologous endogenes and transgenes [30,34,36]. However, they obviously fail to trigger efficient cis-PTGS that would impair viroid replication. In contrast to trans-PTGS, cis-PTGS describes a process where siRNAs trigger silencing of target RNAs that are serving as the source of their production. (2) In tobacco double-transformed with a hairpin- (hp-) PSTVd RNA construct and GUS:PSTVd(+) or GUS:PSTVd(−) fusion transgenes (PSTVd full-length cDNA sequences containing few mutations), vd-siRNAs were abundantly produced but, nevertheless, failed to silence the expression of the GUS:PSTVd transcripts [35]. Similarly, (3) in *Nicotiana* benthamiana protoplasts, co-electroporation of synthetic vd-siRNAs or vd-dsRNAs with PSTVd inoculum did not affect PSTVd replication [23]. Excluding that PSTVd itself may act as a silencing suppressor, the authors suggested that, due to its extensive secondary structure, viroids are resistant to vd-siRNA-mediated cis-PTGS. To test this assumption, transgenes were employed where the GFP was fused to (i) the full-length PSTVd cDNA that forms a rod-shaped secondary structure; (ii) the right-half segment of PSTVd that is predicted to form extensive secondary structures; and (iii) the lower-half PSTVd segment that is predicted to lack extensive secondary structure. These constructs were delivered into *N. benthamiana* protoplasts, together with GFP and PSTVd dsRNAs. While GFP dsRNAs induced efficient silencing of all three constructs, PSTVd dsRNAs only triggered moderate silencing of the third construct [23]. These data suggested that viroid RNA secondary structures, similar to satellite RNAs, indeed, conferred resistance to siRNA-mediated cleavage of mature viroid RNA [23,35].

### 4.2. Examples Where RNA Silencing Efficiently Triggered Viroid Resistance

In contrast to the previous reports described above, more recent data indicated that viroids are not as inherently resistant to trans-PTGS as suggested. (1) Co-inoculation of tomato, gynura and chrysanthemum plants with PSTVd, CEVd, CChMVd and dsRNA corresponding to each viroid, resulted in viroid trans-PTGS in a sequence-specific and temperature/dose-dependent manner [44]. (2) Transgenic tomato plants expressing a hp-PSTVd exhibited trans-PTGS and resistance to PSTVd infection [45]. In contrast to previous approaches, where simultaneous introduction of PSTVd dsRNA and PSTVd inoculum failed to induce viroid silencing [23,44], transgenic plants were used in which hp-derived viroid-specific siRNAs (hp-siRNAs) were present prior to PSTVd inoculation. In this case, the siRNAs should not be called vd-siRNAs as they did not originate from the replicating viroid but from a transgene construct. Interestingly, small RNA deep sequencing revealed that hp-derived siRNAs and vd-siRNAs displayed similar hotspots of accumulation and (+)/(−) polarity distribution patterns [45]. (3) In a grafting approach, transgenic tobacco rootstocks expressing a hp-PSTVd transgene in companion cells under a strong cell-specific promoter, resulted in trans-PTGS and attenuated PSTVd infection in the scions [46]. (4) In PSTVd-infected *N. benthamiana*, 21-nt vd-siRNAs associate with AGO1, AGO2, and AGO3, while 24-nt vd-siRNAs with AGO4, AGO5, and AGO9 [27]. Overexpression of AGO1, AGO2, AGO4 and AGO5 attenuated the levels of replicating PSTVd, suggesting that the mature viroid, or their precursors, are siRNA-targeted [27]. Moreover, these data suggest that under natural conditions, AGOs occupancy may be a limiting factor for efficient vd-siRNAs-mediated viroid silencing. This is reminiscent of a tobacco *in vitro* system, in which overexpression of AGOs was shown to be required to achieve efficient RNA silencing [47]. Finally, (5) co-infection experiments of *Citrus dwarfing viroid* (CDVd) and *Citrus tristeza virus* (CTV) led to elevated viroid titre due to CTV’s silencing suppressors [48], suggesting that viroids could not fully evade RNA silencing under native conditions. Viroids do not encode any suppressors of silencing and replicate in subcellular compartments (nucleus or chloroplast) where vd-siRNAs-mediated PTGS seems to be absent [29]. However, during viroid cell-to-cell movement, they have to cross the cytoplasm where vd-siRNA-mediated PTGS takes place [30]. Obviously, the degree of silencing is not sufficient to tame viroid titres, potentially due to the rapid movement of the molecules and/or the protection of mature viroid RNA molecules by associated proteins.

## 5. Future Perspectives

### 5.1. Artificial Micro RNAs As a Tool for Viroid Silencing

Although potent, siRNA-mediated RNA silencing approaches exhibits major disadvantages. DCL-processing of transgene-derived viroid hp-RNA results in the accumulation of siRNAs that may not only trigger trans-PTGS of the viroid, but also endogene silencing due to off-target effects. It is assumed that vd-siRNAs trigger the development of symptoms [32,33,34,35,36,49,50]. In support of this, tomato plants expressing a hp-PSTVd transgene to generate abundant viroid-specific hp-derived siRNAs exhibited symptoms similar to those of natural PSTVd infections [35]. Another disadvantage of dsRNA/hp-RNA/siRNA-based antiviroid technology is that inverted repeat- (IR-) PTGS and sense- (S-) PTGS are inhibited at low/high temperatures and low/high light intensities [51,52].

In order to avoid off-target effects and environmental dependency of IR-PTGS and S-PTGS approaches, artificial microRNAs (amiRNAs) can be employed [53,54]. In general, miRNAs are processed from POLII-transcribed endogenous miRNA genes (*MIR* genes) [55]. In plants, primary *MIR* gene transcripts (pri-miRNAs) are stabilized by DAWDLE (DDL) and transferred into nuclear D-bodies where they are processed into precursor transcripts (pre-miRNAs) in a process requiring SERRATE (SE), HYPONASTIC LEAVES1 (HYL1) and CAP-BINDING COMPLEX (CBC) [56]. Depending on the plant species pre-miRNAs are ~70- to 2000-nt long stem-loop-structured RNAs that are further processed by DCL1 into mature 21 to 24 nt duplex miRNAs (miRNA:miRNA*) [55]. As DCL endonuclease products and similar to siRNAs, mature miRNA:miRNA* exhibit 3' 2-nt overhangs. Like siRNAs, miRNAs are protected against degradation by SMALL RNA DEGRADING NUCLEASE (SDN) through HEN1 methylation at their 3' ends and are exported into the cytoplasm through HASTY. In the cytoplasm, miRNAs preferentially having a 5' uracil are loaded onto AGO1 and trigger PTGS [55]. AmiRNA design is based on sequences derived from endogenous MIR genes. The endogenous miRNA:miRNA * regions of pri-miRNAs are replaced by artificial miRNA:miRNA*s of which the corresponding amiRNAs are complementary to desired target sequences [53,54]. AmiRNAs have been extensively used to silence genes in *Arabidopsis thaliana* [57], *Oryza sativa* [58], *Solanum melongena* [59], *Physcomitrella patens* [60], and *Chlamydomonas rheinhardtii* [61]. In addition to the silencing of endogenes, amiRNAs have been used to confer resistance to viruses in *A. thaliana* [62], *Nicotiana tabacum* [63] and *Solanum lycopersicum* [64]. While not yet thoroughly tested, it is likely that amiRNAs will also confer resistance against *Pospiviroidae* and *Avsunviroidae*. Optimization of antiviroid amiRNA strategies could be achieved by (1) targeting viroid regions exhibiting low secondary structures to avoid the risk of structure-based insensitivity to PTGS; (2) targeting conserved viroid regions enabling simultaneous resistance to several viroids; (3) non-targeting of viroid sequences that share homology with host genes thereby minimizing off-target effects; and (4) using 22-nt amiRNAs targeting distant regions along the viroid sequence to activate RDR6-mediated amplification of silencing. It has been shown that, in contrast to fully complementary 21-nt sRNAs, 22-nt sRNAs and 21-nt sRNAs containing an asymmetric bulge can trigger transitive silencing [65,66,67,68,69]. Transitive silencing will, however, enhance the probability of off-target effects. Importantly, and in contrast to IR-PTGS/S-PTGS, miRNA/amiRNA-mediated PTGS is temperature-independent [52], thus rendering the amiRNA approach an attractive option for field scale applications.

### 5.2. Additional Silencing Targets

Viroid resistance strategies should not be necessarily confined to siRNA/amiRNA-targeting of only mature viroid and/or its replication intermediates. Host proteins that interact with the viroid and facilitate its replication could also serve as potential silencing targets, provided that they do not interfere with normal plant development. Some examples may include (1) the VIRP1, which, in tomato and tobacco plants, binds the terminal right PSTVd domain to transport the viroid into the nucleus [5,70]; (2) the CUCUMBER PROTEIN 2, which interacts with HSVd [71]; (3) the *Arabidopsis* RIBOSOMAL PROTEIN L5 and transcription factor IIIA, which interact *in vitro* with PSTVd [72]; (4) the avocado chloroplastic protein PARBP33, which interacts with ABSVd *in vivo* and promotes its self-cleavage *in vitro* [73]; (5) the DNA ligase, which is involved in the circularization of PSTVd in tomato [8]; and (6) the chloroplastic tRNA ligase, which is involved in the circularization of *Avsunviroidae* members [14].

### 5.3. Exogenous Application of Silencing Triggers

In view of the regulations for genetically modified organisms (GMOs) valid in several parts of the world, stable or transient expression of nucleic acids inducing PTGS should be considered. In contrast to genome-integrated DNA constructs, application of RNA molecules and introduction of non-integrating, autonomously replicating mini-chromosomes are not under GMO regulations thus far. Exogenously provided RNA molecules (ssRNAs, dsRNAs, siRNAs, amiRNA precursors and amiRNAs) with the potential to elicit RNA silencing can be considered for small scale (mechanical inoculation) and/or large-scale (spraying) antiviroid applications. In this view, bacterial crude dsRNA extracts conferred considerable resistance against *Pepper mild mottle virus* (PMMoV) and *Plum pox virus* (PPV) in *N. benthamiana* [74,75], *Sugarcane mosaic virus* (SCMV) in maize [76], and *Papaya ringspot virus* (PRSV) in papaya plants [77]. In addition to viruses [78], several endogenous targets have been silenced by exogenous application of polynucleotide molecules [79].

### 5.4. Alternative Strategy: Overexpression of Proteins

In addition or as an alternative to viroid and/or host gene silencing, overexpression of host proteins that could affect viroid replication may be useful to diminish viroid titres. Similar to gene silencing, overexpression of host proteins can interfere with normal plant development. (1) With the notable exception of DCL4, the activity of DCL1, 2, and 3 appeared to reduce PSTVd titres in *N. benthamiana* [80]. Thus, overexpression of DCL1, DCL2, and/or DCL3 would probably negatively affect viroid replication; (2) RDRs are most probably not involved in viroid replication. However, RDR1 is induced upon viroid infection [38]. In the case of *Pospiviroidae*, oligomeric (+) replication intermediates localize in the nucleolus [4], where RDR2 is also present [81]. Thus, functions of RDR2 in the lifecycle of *Pospiviroidae* cannot be excluded. Importantly, in *N. benthamiana*, RDR6 activity delays viroid accumulation and precludes PSTVd invasion of meristem tissues [82]. RDR6 may use as template mature viroid RNAs of both families or, in the case of *Pospiviroidae*, also replication intermediates, in the nucleus and/or cytoplasm. Thus, overexpression of at least RDR6 may reduce viroid titres; (3) In *N. benthamiana*, PSTVd-derived siRNAs are loaded onto AGO1, AGO2, AGO3, AGO4, AGO5, and AGO9 [27]. Importantly, transient overexpression of AGO1, AGO2, AGO4, and AGO5 in PSTVd-infected tissue attenuated the level of mature viroid RNAs [27]. Whether AGOs occupancy is a limiting factor in antiviroid defense is not clear, but it is worth mentioning that, in a tobacco *in vitro* system, antiviral defense was facilitated by the overexpression of AGOs [47].

In order to confer viroid resistance through protein overexpression, transgene expression must be stable. Unfortunately, it is commonly observed that overexpressed transgenes frequently become spontaneously self-silenced [83]. Most probably, transgenes facilitate the accumulation of aberrant RNAs (abRNAs) that are devoid of 5' cap and/or 3' polyadenylation tail [84,85]. AbRNAs are potentially deleterious, and are thus eliminated by the RDR-mediated cis-PTGS mechanism [16,86]. However, the employment of transgene constructs containing introns may help to reduce the susceptibility to cis-PTGS [87,88]. AGO1 and DCL1 are regulated by miR168 and miR162, respectively [55]. Thus, artificial miRNA sponges [89] that would specifically sequester miR168 and miR162 could be used to overexpress AGO1 and DCL1. Several approaches that are discussed in this review are currently under investigation in other and our laboratories. Engineering viroid resistance is an exciting field yet it still has a long way to go.
